# Mass spectrometry in ocular drug research

**DOI:** 10.1002/mas.21861

**Published:** 2023-08-02

**Authors:** Eva M. del Amo, Kati‐Sisko Vellonen, Arto Urtti, Tetsuya Terasaki, Anam Hammid, Paavo Honkakoski, Seppo Auriola

**Affiliations:** ^1^ School of Pharmacy University of Eastern Finland Kuopio Finland; ^2^ Division of Pharmaceutical Biosciences, Faculty of Pharmacy University of Helsinki Helsinki Finland

**Keywords:** drug analysis, LC‐MS/MS, mass spectrometry, MSI, ocular pharmacokinetics, proteomics, quantitation, spatial distribution

## Abstract

Mass spectrometry (MS) has been proven as an excellent tool in ocular drug research allowing analyzes from small samples and low concentrations. This review begins with a short introduction to eye physiology and ocular pharmacokinetics and the relevance of advancing ophthalmic treatments. The second part of the review consists of an introduction to ocular proteomics, with special emphasis on targeted absolute quantitation of membrane transporters and metabolizing enzymes. The third part of the review deals with liquid chromatography–MS (LC‐MS) and MS imaging (MSI) methods used in the analysis of drugs and metabolites in ocular samples. The sensitivity and speed of LC‐MS make simultaneous quantitation of various drugs and metabolites possible in minute tissue samples, even though ocular sample preparation requires careful handling. The MSI methodology is on the verge of becoming as important as LC‐MS in ocular pharmacokinetic studies, since the spatial resolution has reached the level, where cell layers can be separated, and quantitation with isotope‐labeled standards has come more reliable. MS will remain in the foreseeable future as the main analytical method that will progress our understanding of ocular pharmacokinetics.

**Abbreviations**
DESI: desorption electrospray ionization
DIA: data‐independent acquisition
ESI: electrospray
FA: formic acid
FFPE: formalin‐fixed paraffin‐embedded
FT‐ICR: Fourier‐transform ion cyclotron resonance
H‐PCT: heated pressure cycling technology
IHC: immunohistochemistry
LA‐ICP‐MS: laser ablation inductively coupled plasma mass spectrometry
LLE: liquid‐liquid extraction
LMD: laser micro‐dissection
LTQ: linear ion quadrupole
MALDI: matrix‐assisted laser desorption ionization
MALDI‐2: MALDI with a second laser for postionization of the sample plume generated with the first laser
MRI: magnetic resonance imaging
MSI: mass spectrometric imaging
NanoSIMS: nano secondary ion mass spectrometry
OCT: optical coherence tomography
PASEF: parallel accumulation−serial fragmentation
PPT: protein precipitation
PRM: parallel reaction monitoring
prm‐PASEF: parallel reaction monitoring‐parallel accumulation−serial fragmentation
Q‐TOF: quadrupole time of flight
RMS: root mean square
RPE: retinal pigment epithelium
SIMS: secondary ion mass spectrometry
SPE: solid phase extraction
SRM: selective reaction monitoring
TBME: tert‐butylmethylether
TIC: total ion current
timsTOF: trapped ion mobility spectrometry‐time of flight
xMD: expression microdissection

## 1 INTRODUCTION

Overall, 2.2 billion people suffer from visual impairment (WHO, 2019), and the number of patients suffering from age‐related macular degeneration (AMD), diabetic retinopathy, and glaucoma were about 196, 146, and 76 million by 2020, respectively (WHO, 2019). Efficient ocular drug development is highly in demand due to the rapid increase in the number of patients.

Interest in ocular pharmacokinetics and drug delivery has grown remarkably after the successful launch of biologics as treatment for neovascular AMD (Chakravarthy et al., 2022). The number of publications in ocular drug delivery increased 12‐fold from 2000 to 2020, and industrial interest has also grown much beyond specialized companies with ocular drug development.

Improved research methods are needed to solve the challenges in ocular drug delivery. For example, topical administration results in low ocular bioavailability (0.1%–5%) to the anterior parts of the eye, like aqueous humor, and only negligible delivery to the posteriorly located retina and choroid that are the main targets in ocular drug discovery and development (del Amo, 2022; Fayyaz et al., 2021; Naageshwaran et al., 2021, 2022; Salminen & Urtti, 1984). Similarly, systemic drug administration yields only low drug levels in the ocular tissues because blood‐ocular barriers limit access of drugs into the eye (Ramsay et al., 2019; Vellonen et al., 2016). Small molecules with adequate lipophilicity can cross the tight ocular membranes at the ocular surface (corneal and conjunctival epithelium) and blood‐ocular barriers (tight vascular walls in the iris, ciliary muscle and retina, and retinal pigment epithelium‐RPE‐and inner iris‐ciliary epithelium) (del Amo et al., 2017). Hydrophilic compounds, protein‐bound compounds and biologics have low, usually inadequate, absorption and distribution into the eye from eyedrops and blood circulation, respectively. For these reasons, invasive intravitreal injections, directly into the eye, have become a widely used method in retinal and choroidal drug treatment (del Amo et al., 2017). Quantitation of drug concentrations in the eye has become an important and timely methodology because the improved quantitative understanding of ocular pharmacokinetic processes is very useful in drug development. New drugs are needed as most retinal diseases are still without effective treatments. Also, better drug delivery systems are required to enable effective, less invasive, and long‐acting drug treatments (Kang‐Mieler et al., 2020).

Sampling from the eye is difficult because only tear fluid can be continuously sampled (Lee & Robinson, 1979). The aqueous humor can be withdrawn with a needle and syringe a few times at long sampling intervals of many days. Thus, ocular pharmacokinetic studies involve the use of many laboratory animals that must be killed before eye tissues can be taken for the analyzes. Rabbit is the most widely used animal model, because of its physiologic and anatomic similarities, for example, its tissue sizes are comparable to those in humans (del Amo & Urtti, 2015). It is noteworthy that mice and rats are widely used in early pharmacological studies, but rodent eyes are not good models in ocular drug delivery studies, because pharmacokinetics in so small eyes differs remarkably from human eyes, dissection of the small tissues is difficult, and quantitative drug analytics from tiny samples is challenging. Obviously, pharmacokinetic studies in human eyes are hindered by ethical considerations. Therefore, human ocular pharmacology is limited mostly to drug response studies, but pharmacokinetics and drug delivery have only rarely been quantitated (del Amo & Urtti, 2015).

In rabbit eyes, the tissues are small (range of 0.05–0.3 g), but in the rodent eyes even smaller by at least one order of magnitude. In rabbits, after topical eye drop instillation, concentrations of small molecule drugs show a wide range (1–10^6^ pmol/g tissue) depending on the properties and location of the tissue in the eye (Fayyaz et al., 2021). Furthermore, ocular tissues vary in terms of sample handling as some tissues are liquid‐like (aqueous humor, vitreous) and some are rigid (e.g., sclera). Thus, ocular pharmacokinetics is a challenging field for quantitative pharmaceutical analysis.

Ocular pharmacokinetics studies have been performed with various analytical methods, such as high‐performance liquid chromatography (HPLC) (Sparado & Pappalardo, 2015), enzyme‐linked immunosorbent assay (ELISA) (Bakri et al., 2007; Miyake et al., 2010), liquid scintillation counting (Conrad & Robinson, 1980; Sieg & Robinson, 1974), radiochemical imaging (Luaces‐Rodríguez et al., 2020; Schmitt et al., 2019), magnetic resonance imaging (MRI) (Li et al., 2008), and fluorophotometry (Joshi et al., 1996; McCarey, 2011). However, these methods have some drawbacks. In general, HPLC does not have sufficient sensitivity to analyze low drug concentrations in small eye tissues. ELISA is mainly used for biological drugs, but it does not inform about the potential changes in the drug structure (e.g., amino acid modifications, metabolism, and degradation). Liquid scintillation counting of ^3^H‐ and ^14^C‐labeled drugs was used widely in the past due to its excellent sensitivity, easy sample preparation, and wide range of linearity. However, the current low availability of radiolabeled drugs limits the use of this method. Also, metabolite analyzes require chromatographic separation (Salminen & Urtti, 1984). More recently radiochemical imaging (positron emission tomography, single‐photon emission computed tomography) has been developed, but the instruments are rarely applicable to rabbit eyes, the spatial resolution does not allow the detection of individual tissues in rodent eyes, and metabolic processes are not easily resolved (Schmitt et al., 2019). The short half‐life of the isotopes (^99m^Tc, ^119^In) is another serious limitation in studies of long‐acting formulations. MRI provides an excellent spatial resolution but suffers from poor sensitivity and narrow dynamic range. Fluorescence methods have some advantages, such as high sensitivity, imaging possibilities, and availability of the dyes. Because cornea, aqueous humor, lens, and vitreous humor are transparent, noninvasive detection of fluorescently labeled biologics and particles is possible if the excitation and emission wavelengths are compatible with the Fluorotron device (Sadeghi et al., 2022). Obviously, labeling with fluorophores changes the molecular properties of small molecules too much and leads to wrong pharmacokinetic conclusions.

Mass spectrometry has emerged as the method of choice in ocular pharmacokinetic studies, as it enables the analyzes of drugs, metabolites, biomarkers, and drug targets. Pharmacokinetic studies require absolute quantitation of the compounds because that is the only way to obtain pharmacokinetic parameters and understand the mass balance of the compounds between various ocular tissues. Sampling challenges include variations between tissue matrices from that range from simple liquids such as aqueous humor to much harder tissues like cornea. Small sample sizes and low concentrations represent another challenge. On the other hand, mass spectrometry offers many possibilities that have been rarely utilized, such as the simultaneous quantitation of various drugs (Fayyaz et al., 2021) and metabolites (del Amo et al., 2022). Imaging mass spectrometry is able to provide rich spatial data on various compounds simultaneously, potentially taking the understanding of ocular drug delivery to high spatial resolution and linking pharmacokinetics with drug responses (Liu et al., 2020; Wang et al., 2019).

**Figure 1:**
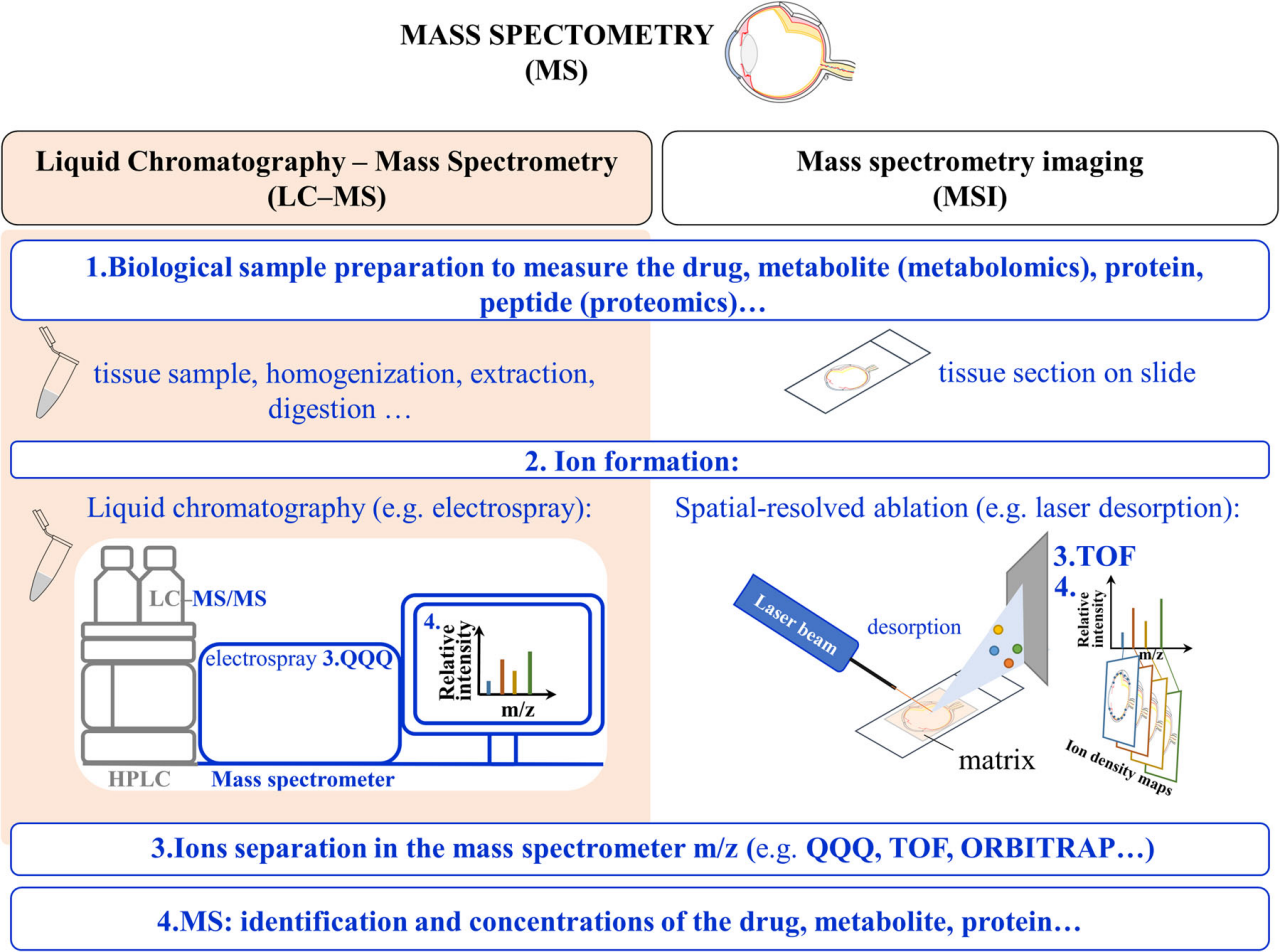
Schematic representation of the mass spectrometry workflow applied in ophthalmic research. [Color figure can be viewed at wileyonlinelibrary.com]

A schematic representation of mass spectrometry workflow in ocular research is presented in Figure 1. With this review we aim to describe how mass spectrometry is used to study the ocular expression of drug metabolizing enzymes and transporter proteins important for drug development, give examples of how liquid chromatography–mass spectrometry (LC‐MS) is used in pharmacokinetic studies and give an introduction to the possibilities of mass spectrometry imaging for spatial analysis of the eye.

## 2 THE ANATOMY OF THE EYE, ROUTES OF ADMINISTRATION, AND PHARMACOKINETICS

**Figure 2:**
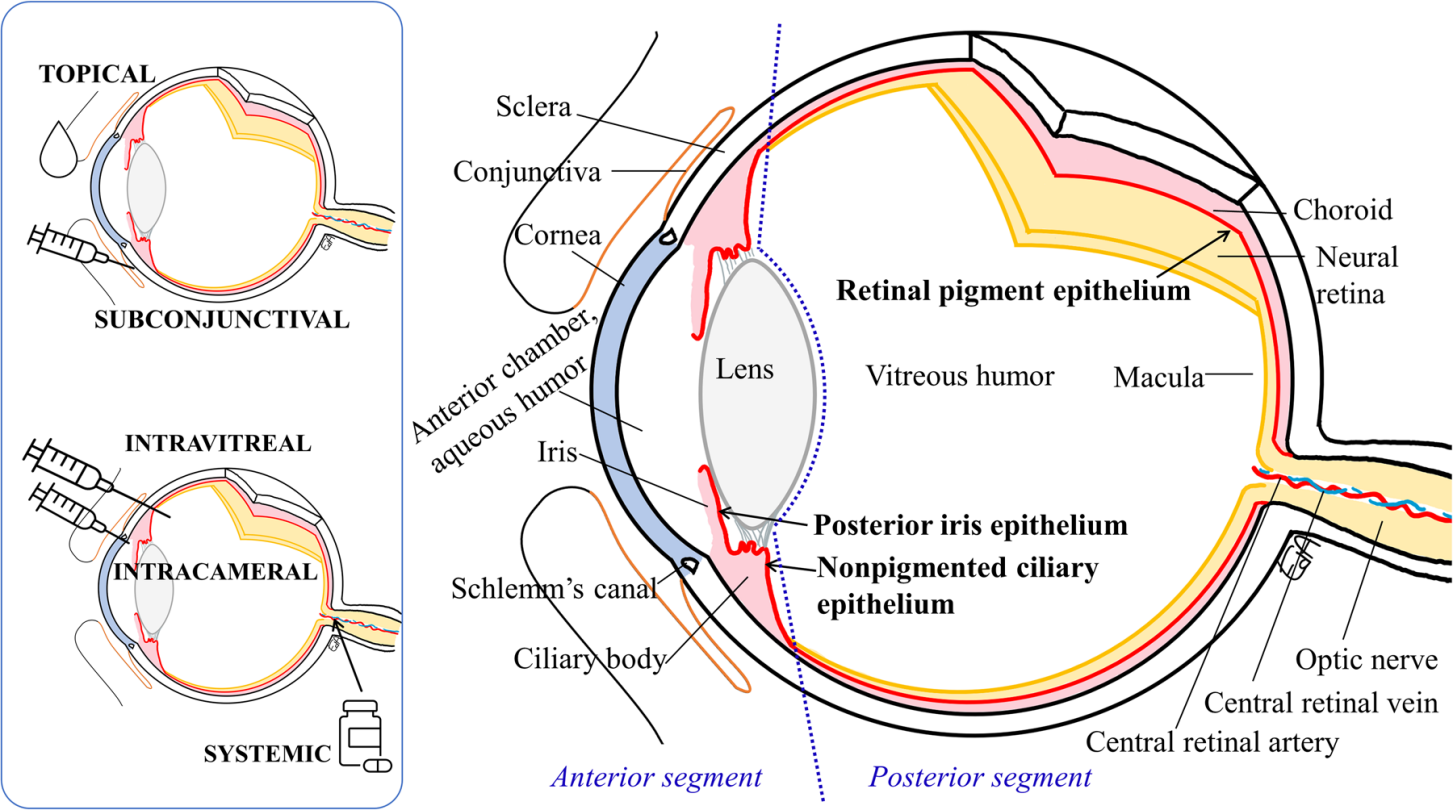
Scheme of ophthalmic drug administration routes (left), and the anatomy of the eye on the right, showing the drug permeation barriers. [Color figure can be viewed at wileyonlinelibrary.com]

The eye is a well‐protected organ, with effective physical and biochemical mechanisms that limit the ocular distribution of xenobiotics. In the anterior segment of the eye, the corneal epithelium with its tight intercellular junctions limits the absorption of hydrophilic small molecules and macromolecules (Figure 2) (Huang et al., 1989; Ramsay et al., 2018). Tight junctions are present also in the iris and ciliary muscle capillaries, and in the posterior iris and nonpigmented ciliary epithelia (Figure 2), which constitute the blood‐aqueous barrier (Raviola, 1974, 1977). In the posterior segment of the eye, the blood‐retinal barrier includes the retinal vessels and RPE (between the retina and the choroid) (Figure 2) (Cunha‐Vaz & Maurice, 1969; Pitkänen et al., 2005; Raviola, 1977). Regardless of the route of administration, distribution of drugs to the eye is always somehow restricted.

After topical drug administration, drainage of the excess volume and increased tear outflow occurs rapidly, with most of the drug cleared into the nasolacrimal system and further into the systemic circulation. Most of the dose absorbed via conjunctiva will reach the systemic circulation as well (Urtti & Salminen, 1993). Only less than 5% of the dose reaches the anterior chamber (Fayyaz et al., 2021). From the aqueous humor, the drug is eliminated to the Schlemm canal or vascularized iris‐ciliary body, eventually reaching blood circulation. Topical administration is the most widely used route of drug administration in the treatment of anterior segment diseases, such as elevated intraocular pressure and inflammatory conditions. However, it is not a suitable route for drug delivery to treat posterior segment illnesses, such as AMD and diabetic retinopathy since the drug exposure in the posterior tissues is low after topical administration (Fayyaz et al., 2021; Holló et al., 2006; Urtti et al., 1990). Nevertheless, this matter is under much debate in the community research (del Amo, 2022; Löscher et al., 2022; Rodrigues et al., 2018).

Posterior eye segment diseases are typically treated using intravitreal injections, with the first intravitreal formulation approval by the Food and Drug Administration (FDA) in 1996 (Martin, 2018; Smith et al., 1992) (Figure 2). Antiangiogenic biological drugs are intravitreally injected to treat AMD and diabetic retinopathy (Brown et al., 2006; Heier et al., 2012; Rosenfeld et al., 2005, 2006) These drugs (such as bevacizumab, ranibizumab, and aflibercept) have hydrodynamic radii much bigger than 2 nm. Since they do not cross blood‐ocular barriers their elimination takes place anteriorly (del Amo et al., 2017; Caruso et al., 2020; Shatz et al., 2016) and their half‐lives in the vitreous are in the range of days, whereas small molecule drugs have half‐lives in the range of hours (del Amo et al., 2015).

Systemic administration is only rarely used to treat ocular diseases because only a small fraction of the systemic drug reaches the ocular tissues (Mysore et al., 2011; Vellonen et al., 2016). The other routes of administration, such as subconjunctival and intracameral routes, are sometimes used for drug delivery to the anterior segment (Figure 2).

Regarding ocular drug distribution, cornea epithelium may act as depot tissue for lipophilic drugs after topical and intracameral administrations while lenticular drug partitioning tends to be low (del Amo et al., 2022; Fayyaz et al., 2021; Lee & Robinson, 1979; Naageshwaran et al., 2022). However, some drug may distribute to the lens after intravitreal injection (del Amo et al., 2022), probably into the outermost lens layers as observed in an in vitro incubation study with porcine lens (Heikkinen et al., 2018). To our knowledge limited information is available for transporter protein expression in the lens epithelium, Zhang and collaborators found mdr1a, mrp1, mrp5, bcrp, mct1, and mct4 by proteome‐transcriptome analysis in mouse lens epithelium, only reported (Zhao et al., 2019). Another relevant aspect is the ocular pigment, melanin binding can also increase the intracellular drug accumulation in the ocular pigmented tissues of the uveal tract, with impact on drug biodistribution and pharmacodynamics (Rimpelä et al., 2020; Shibata et al., 1988, Urtti et al., 1984).

There is only limited information about the role of transporters, enzymes, and receptors on drug distribution into the eye or on drug elimination across those barriers. Mass spectrometry will be the key technology in exploring the functions of the ocular barriers and their impact on the functionality of potential ocular drug treatments. Different ocular diseases have distinct cellular and tissue targets necessitating accurate drug and target protein analytics in specific ocular tissues, including various cell types in the retina.

## 3 ANALYSIS OF PROTEINS IN OCULAR SAMPLES

One of the most significant advantages of proteomics is that global proteomics can provide direct evidence of relevant transporter/receptor/enzyme proteins expression in the target region, whereas quantitative targeted proteomics enables us to evaluate differential capacities of specific proteins. These are useful features in ocular drug research. Global proteomics of ocular tissues after exposure to drug or drug candidate provides information about drug efficacy and safety. Global and quantitative target proteomics give rational insights into pharmacokinetics, pharmacodynamics, and toxicokinetics by analyzing protein level in the target region, posttranslational modification of relevant proteins, protein–protein interaction, and protein complex formation (Huttunen et al., 2022; Ohtsuki et al., 2011). Quantitative proteomics analyzing stable isotope labeled serum proteins or protein drugs in the aqueous humor after systemic administration has proven to be useful way to evaluate integrity of the blood‐aqueous humor and investigate the possible impact of ocular neovascularization on the barrier (Liu et al., 2018). Global and quantitative proteomics for the analysis of aqueous humor in the patients showed sufficient sensitivity for identifying several proteins relevant to the disease such as cornea endothelial decomposition (Peng et al., 2023). Finally, protein expression and activity in the target region can be utilized in pharmacokinetic models by incorporating the protein levels and the intrinsic protein activity from in vitro studies (Uchida et al., 2011; Uchida, Ohtsuki, et al., 2014; Uchida, Wakayamma, et al., 2014).

### 3.1 Workflow of receptor, transporter, and enzyme proteins quantification in the ocular tissues

**Figure 3:**
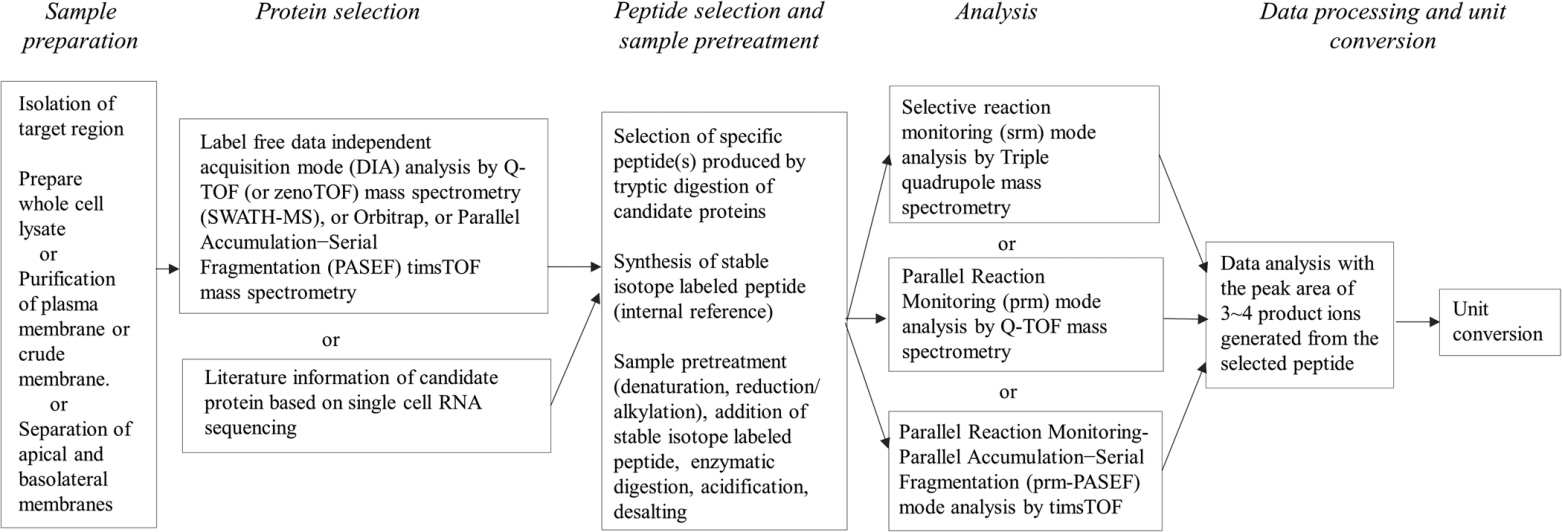
Workflow for quantification of transporters, receptors, and enzymes.

There are five steps for protein quantification by mass spectrometry, summarized in Figure 3.

*Sample preparation*: First, target tissues from the eye must be isolated, for example, corneal epithelium, RPE, or retinal capillary endothelial cells. The RPE, a monolayer of cuboidal cells, can be gently collected from the anterior part of the eyeball with a small paintbrush (Zhang et al., 2017). Although several methods have been proposed for the isolation of RPE (Amirpour et al., 2014), it is a key issue to use the method without enzymatic digestion for dissection (Zhang et al., 2017). Depending on the purpose and the sample quantity, whole cell lysate, crude plasma membrane or plasma membrane fractions of the isolated tissue will be prepared. If there is an adequate amount of plasma membranes, the apical and basolateral membrane fractions can be separated for the analysis.

*Protein selection*: Next, candidate proteins for quantitation should be selected. There is no general rule for the selection. It is possible to use literature information such as functional analysis, messenger RNA (mRNA) analysis, western blot analysis, or single RNA sequencing for selection guidance. The selection depends also on the sensitivity of the mass spectrometric method. The traditional global proteomics with data‐dependent acquisition (DDA) mode analysis may not give sufficient assay sensitivity for low‐abundance proteins. In that case, it may be better to use data‐independent acquisition (DIA) mode, such as SWATH‐MS for the protein selection (Gillet et al., 2012). If recently developed ultra‐highly sensitive mass spectrometry instruments such as Zeno trap pulsing mass spectrometer, ZenoTOF (Wang et al., 2022) or trapped ion mobility mass spectrometer, timsTOF Pro (Meier et al., 2018, 2020) are available, the DIA mode analysis would give even higher numbers of potential candidate proteins for quantification. However, the result of DIA significantly depends on the quality of the database containing the parent peptide, product ions, and retention time. There are also limitations in the suitability of DIA for the identification and quantification of posttranslationally modified peptides.

*Peptide selection and sample pretreatment*: As the membrane proteins contain hydrophobic transmembrane domain, it has to be dissolved for the enzymatic digestion which is different from the standard protocol of proteomics for water‐soluble proteins. 6 M urea is often used for the solubilization (Kamiie et al., 2008; Uchida et al., 2013). The sample is digested by lysyl endopeptidase (LysC), and then with trypsin. After this it is crucial to find specific proteotypic peptides that are produced in the enzymatic digestion with good yield and good analytical properties. Global proteomics can often be used to find these candidate peptides for targeted quantitation. However, even if there is no prior information about the peptides, the candidate peptides could be selected by several criteria (Kamiie et al., 2008; Uchida et al., 2013). If there are peptides with the same amino acids, but different sequence, it would be very difficult to distinguish between various peptides with the same amino acids, but different sequences. However, careful selection of the product ions could help to distinguish between different peptide(s) (Ohtsuki et al., 2012). Most peptides are stable during the enzymatic digestion, but there is always possibility of asparagine deamidation and aspartate isomerization (Yang & Zubarev, 2010). As proteins could be artificially oxidized during the sample preparation, typical reactions including carbonylation of lysine, proline, and arginine, oxidation of cysteine and methionine and hydroxylation of proline, it would be better to exclude these peptides from the data analysis.

For quantitative target proteomics stable isotope‐labeled peptide analogs are synthetized and added to the sample as an internal reference. Stable isotope‐labeled peptide, an internal reference, is crucial for the correction of solubility, adsorption, and ionization efficiency of the peptide produced from target protein. Nevertheless, multiple peptides from the same protein would give different quantitative results which could be attributed to the efficiency of digestion by trypsin. To minimize the inefficient cleavage, the following criteria should be considered for the peptide selection, (1) excluding continuous sequence of arginine or lysine residues (RR, KK, RK, KR), (2) excluding a proline residue at the C‐terminal side of an arginine or lysine residue (RP or KP) in the digestion region, (3) excluding a transmembrane region (Kamiie et al., 2008; Uchida et al., 2013).

*Analysis*: The sample is injected onto the C18 column of the liquid chromatogram (LC) connected to the triple quadrupole mass spectrometer (QqQ) by employing the selective reaction monitoring (SRM) mode (Kamiie et al., 2008) or quadrupole time of flight mass spectrometer (Q‐TOF) by the parallel reaction monitoring (PRM) mode (Zhang et al., 2017). The latest timsTOF Pro (Bruker) can achieve sensitive target quantification with ultrahigh resolution by the parallel reaction monitoring‐parallel accumulation−serial fragmentation (prm‐PASEF) mode analysis (Brzhozovskiy et al., 2022).

*Data processing*: Three to four transitions per peptide that correspond to high‐intensity product ions are selected for the native peptides from sample and stable isotope‐labeled internal reference. To obtain reliable results, it would be crucial to analyze the data with at least three transitions per peptide (Kamiie et al., 2008; Uchida et al., 2013). Nevertheless, it would be possible to indicate the value analyzed by one or two transitions as a preliminary data if you would provide the number of transitions used in the result.

*Unit conversion*. The result of protein quantification by mass spectrometry is usually presented as fmol/μg protein (Huttunen et al., 2022). If the protein quantification is performed for the whole cell lysate, the content of plasma membrane or microsomes would be constant among preparations. However, when the plasma membrane or microsome are prepared and quantified, it is not possible to exclude the possibility of inter‐laboratory differences in purity of plasma membranes or microsomes. Such differences would affect the results that are presented as fmol/μg protein (Huttunen et al., 2022). Assuming the recoveries of marker proteins and targeted proteins are the same, it is possible to convert the unit from fmol/μg protein to fmol/wet tissue weight (Uchida et al., 2020a). The selected marker proteins should be abundantly expressed in the plasma membranes or microsomes. This unit conversion is helpful in minimizing the impact of sample preparation impurities and in understanding the protein activity in the ocular tissue. As summarized in Table 1, fmol/cm^2^ of surface area of the plasma membrane (Uchida, Yagi, et al., 2020) and fmol/whole region (Uchida, Goto, et al., 2020) could be also useful.

**Table 1:** Units of transporter, receptor, and enzyme levels.

Purpose	Unit of protein level
Initial data of transporter/receptor/enzyme protein quantification	fmol per μg protein of sample
Evaluation of maximum capacity of transport or enzyme activity in different regions or different pathophysiological conditions	fmol per g wet tissue weight
Evaluation of active transport activity affecting to the unbound concentration gradients between extracellular and intracellular space	fmol per cm^2^ of surface area of the plasma membrane
Evaluation of maximum capacity of transport or enzyme activity in different regions or different pathophysiological conditions	fmol in the whole region

### 3.2 Proteomics of transporters: The blood‐retinal barrier

*Comparison of inner (iBRB) and outer blood‐retinal barrier (oBRB)*: Retina is protected from the xenobiotics in the systemic bloodstream by two BRBs. The iBRB consists of the retinal vascular endothelium, whereas the oBRB is based on RPE. The BRB plays a crucial role in regulating the exchange of nutrients and drugs between blood and the retinal tissue. As the cells in both iBRB and oBRB are connected with tight junctions, transcellular permeation is the only effective pathway for substrate exchange. Therefore, it is important to clarify the transporter expression to understand the physiological and pharmacokinetic role of the BRB.

Sixteen ABC transporter and 13 SLC transporter proteins in the crude membrane fractions of isolated retinal capillaries and isolated RPE have been from quantified porcine eyeballs. As summarized in Table 2 (Zhang et al., 2017), five transporter proteins were detected at the inner BRB, that is, breast cancer resistance protein (BCRP/*ABCG2*), multiple drug resistance 1 (MDR1/*ABCB1*), monocarboxylate transporter 1 (MCT1/*SLC16A1*), glucose transporter 1 (GLUT1/*SLC2A1*) and sodium‐potassium ATPase (Na^+^/K^+^‐ATPase). The transporter protein expression of the porcine iBRB correlated with the expression levels in the porcine brain capillary endothelial cells (*R* ^2^ = 0.89) (Zhang et al., 2017). Interestingly, the porcine iBRB expresses BCRP dominantly, while oBRB shows eight times lower levels. BCRP protein level in the iBRB is 2.6‐fold higher than that of MDR1 (Table 2). Thus, BCRP in the iBRB is likely to serve as an efflux transporter of phototoxic endogenous and exogenous substrates, such as pheophorbide A, fluoroquinolone antibiotics, and protoporphyrin IX dominantly (Zhang et al., 2017).

**Table 2:** Comparison of transporter protein levels in the crude membrane fraction between retinal capillary and retinal pigment epithelium in pig modified from (Zhang et al., 2017).

		Protein expression level (fmol/μg protein)	
Protein/gene name		Retinal capillary (inner BRB) (mean ± SD)	Retinal pigment epithelium (outer BRB) (mean ± SD)
Breast cancer resistance protein	BCRP/*ABCG2*	22.8 ± 0.5	2.76 ± 0.55
Multidrug resistance 1	MDR1/*ABCB1*	8.70 ± 0.40	2.01 ± 0.06
Monocarboxylate transporter 1	MCT1/*SLC16A1*	4.83 ± 0.43	2.71 ± 0.39
Glucose transporter 1	GLUT1/*SLC2A1*	168 ± 5	56.6 ± 5.0
Sodium‐dependent multivitamin transporter	SMVT/*SLC5A6*	ULQ (<0.248)	0.378 ± 0.091
Organic anion‐transporting polypeptide 3A1	OATP3A1/*SLCO3A1*	ULQ (<0.297)	0.234 ± 0.023
Multidrug resistance‐associated protein 1	MRP1/*ABCC1*	ULQ (<0.955)	1.03 ± 0.11
Sodium‐potassium ATPase	Na^+^/K^+^ ATPase	53.7 ± 2.0	60.7 ± 4.6

*Note*: “ULQ” represents under the limit of quantification. The values (fmol/μg protein) of the limit of quantification were shown in parentheses.

*Comparison of in vitro cultured models of human retinal pigment epithelium (ARPE19 and hfRPE)*: Cultured cell models of RPE cells, such as human fetal RPE (hfRPE), ARPE19, D407, and h1RPE have been used in eye research, but only limited information on transporter expression at protein level has been available. Therefore, Pelkonen et al. (2017) quantified transporter proteins in hfRPE and ARPE19 cells. As shown in Table 3, multidrug resistance‐associated protein 1 (MRP1/*ABCC1*), multidrug resistance‐associated protein 5 (MRP5 *ABCC5*), GLUT1, 4F2 cell‐surface antigen heavy chain (4F2hc/*SLC3A2*), BCRP, sodium‐and chloride‐dependent taurine transporter (TAUT/*SLC6A6*), cationic amino acid transporter 1 (CAT1/*SLC7A1*), large neutral amino acids transporter small subunit 1 (LAT1/*SLC7A5*), and multidrug and toxin extrusion protein 1 (MATE1/*SLC47A1*) proteins were detected in both cell lines within four times differences. Proton‐coupled folate transporter (PCFT/*SLC46A1*) was detected in both cell lines, while the expression in hfRPE was over 4‐fold higher than that of ARPE19. MCT1, monocarboxylate transporter 4 (MCT4/*SLC16A3*), multidrug resistance‐associated protein 4 (MRP4/*ABCC4*), and Na^+^/K^+^ ATPase in the ARPE19 were over 4‐fold greater than those of the hfRPE. ATP‐binding cassette sub‐family C member 10 (MRP7/*ABCC10*), organic anion transporter 2 (OAT2/*SLC22A7*), and reduced folate transporter (RFC1/*SLC19A1*) proteins were identified in the hfRPE cells, while were below the limit of quantification in ARPE19 cells. Overall, transporter protein levels in the ARPE19 cells are correlated well with those of hfRPE, but not identical (Table 3) (Pelkonen et al., 2017). ARPE19 has been often used as in vitro RPE model, whereas further studies are necessary for establishing the in vitro‐in vivo extrapolation of drug transport across the human RPE such as (1) quantify the transporter proteins in the plasma membrane purified from RPE in human and (2) link the transporter concentrations to their activity in the cells.

**Table 3:** Comparison of transporter protein levels in the plasma membrane fractions between hfRPE and ARPE19 modified from (Pelkonen et al., 2017).

	Protein/gene name	hfRPE (mean ± SEM) (*n* = 3) (fmol/μg protein)	ARPE19 (mean ± SEM) (*n* = 5) (fmol/μg protein)	Fold difference hfRPE/ARPE19
Multidrug resistance‐associated protein 1	MRP1/*ABCC1*	1.36 ± 0.41	4.00 ± 2.68	0.34
Multidrug resistance‐associated protein 4	MRP4/*ABCC4*	0.0995^b^	1.59 + 0.53	0.0625
Multidrug resistance‐associated protein 5	MRP5/*ABCC5*	0.789 ± 0.004	1.10^a^	0.716
Multidrug resistance‐associated protein 7	MRP7/*ABCC10*	0.782^b^	ULQ (<0.113)	>6.92
Glucose transporter 1	GLUT1/*SLC2A1*	337 ± 44	205 ± 85	1.64
4F2 cell‐surface antigen heavy chain	4h2hc/*SLC3A2*	3.81 ± 0.56	12.5 ± 2.9	0.306
Sodium‐and chloride‐dependent taurine transporter	TAUT/*SLC6A6*	0.499 ± 0.046	0.441 ± 0.175	1.13
Cationic amino acid transporter 1	CAT1/*SLC7A1*	2.26 ± 0.07	1.65 ± 0.63	1.37
Large neutral amino acids transporter small subunit 1	LAT1/*SLC7A5*	0.721 ± 0.540	2.34 ± 0.51	0.308
Monocarboxylate transporter 1	MCT1/*SLC16A1*	3.14 ± 0.96	15.1 ± 5.3	0.209
Monocarboxylate transporter 4	MCT4/*SLC16A3*	6.91 ± 0.61	35.2 ± 13.7	0.196
Reduced folate transporter	RFC1/*SLC19A1*	1.20 ± 0.46	ULQ (<0.650)	>1.85
Organic anion transporter 2	OAT2/*SLC22A7*	0.207^b^	ULQ (<0.250)	>1.01
Proton‐coupled folate transporter	PCFT/*SLC46A1*	4.91 ± 1.20	0.588^c^	8.35
Multidrug and toxin extrusion protein 1	MATE1/*SLC47A1*	0.851 ± 0.092	1.58 ± 0.46	0.537
Sodium‐potassium ATPase	Na^+^/K^+^ ATPase	12.2 ± 3.1	59.9 ± 26.5	0.203

*Note*: The protein expression levels were calculated as the average of 3 or 4 MRM/SRM transitions. ULQ: represents the values of the quantification limit (fmol/μg protein).

^a^ The expression levels were calculated as an average of 2 quantitative values obtained from two SRM/MRM transitions.

^b^ The expression levels were calculated as the average values obtained from two samples.

^c^ The expression levels were calculated from only a single SRM/MRM transition due to high noise levels at the other transitions.

*Comparison of transporter expression in the apical and basolateral plasma membranes of cultured human fetal retinal pigment epithelial cells (hfRPE)*: The plasma membrane transporters of the RPE cells may contribute to the inward‐directed transport from the choroid to the subretinal space, and/or the outward transport from the subretinal space into the choroid. As the direction of transporter function is important for the understanding of physiological and pharmacological role of RPE transporters, it is important to clarify the localization of transporters in the RPE, that is, apical and/or the basolateral plasma membranes. The apical and basolateral membrane fractions from differentiated hfRPE monolayers were separated using the nitrocellulose peeling method that was combined with sucrose density gradient centrifugation (Fong‐ngern et al., 2009) and quantified transporter proteins.

As summarized in Table 4 (Hellinen et al., 2019), the protein levels of MDR1, LAT1, and MCT1 in the apical membrane fraction were more than 1.5‐fold greater than those of basolateral membrane fraction. Based on the immunohistochemical analyzes, the selective apical membrane expression of MDR1, LAT1, and MCT1 proteins were confirmed. As bestrophin 1, an RPE‐specific basolateral marker protein, was selectively expressed in the basal plasma membrane, the polarized expression of plasma membrane proteins in hfRPE was confirmed (Hellinen et al., 2019). Interestingly, MRP1 protein had lateral membrane localization in the hfRPE cells (Hellinen et al., 2019). When SWATH‐MS analysis was used for the apical and basolateral plasma membranes of hfRPE, it was found that the protein level of putative sodium‐coupled neutral amino acid transporter 7 (SNAT7/*SLC38A7*) and riboflavin transporter (RFT3/*SLC52A2*) were enriched on the basolateral membrane fraction. Table 4 shows various transporters with less than 1.5‐fold expression difference between apical and basolateral plasma membranes. Presumably, some influx transporters could be used as drug delivery pathway from blood circulation to the RPE or neural retina.

**Table 4:** Comparison of transporter protein levels in the hfRPE cells between apical and basolateral plasma membranes (Hellinen et al., 2019).

Name of transporter protein	Abbreviation protein/gene	Basolateral plasma membrane (fmol/μg protein),^a^ (mean ± SEM)	Apical plasma membrane (fmol/μg protein),^a^ (mean ± SEM)	*p*	Expression ratio, apical/basolateral, mean	Localization^b^
Multidrug resistance 1	MDR1/*ABCB1*	0.201 ± 0.029^c^	0.392 ± 0.051^c^	0.0172	1.95	Apical enrichment
Multidrug resistance‐associated protein 1	MRP1/*ABCC1*	2.41 ± 0.16	2.82 ± 0.31	0.281	1.17	Equal abundance
Multidrug resistance‐associated protein 4	MRP4/*ABCC4*	0.205 ± 0.013	0.197 ± 0.01	0.645	0.961	Equal abundance
Multidrug resistance‐associated protein 5	MRP5/*ABCC5*	0.586 ± 0.038	0.827 ± 0.106	0.0771	1.41	Equal abundance
Glucose transporter 1	GLUT1/*SLC2A1*	981 ± 60^c^	1141 ± 84^c^	0.173	1.16	Equal abundance
4F2 cell‐surface antigen heavy chain	4F2hc/*SLC3A2*	10.9 ± 1.7	14 ± 1.6	0.237	1.28	Equal abundance
Sodium‐and chloride‐dependent taurine transporter	TAUT/*SLC6A6*	0.298 ± 0.071	0.238 ± 0.053	0.523	0.799	Equal abundance
Cationic amino acid transporter 1	CAT1/*SLC7A1*	3.4 ± 0.21	3.59 ± 0.59	0.770	1.06	Equal abundance
Large neutral amino acids transporter small subunit 1	LAT1/*SLC7A5*	1.09 ± 0.17^c^	1.87 ± 0.33#	0.0815	1.72	Apical enrichment
Monocarboxylate transporter 1	MCT1/*SLC16A1*	9.96 ± 0.66	16 ± 2.06	0.0322	1.61	Apical enrichment
Monocarboxylate transporter 3	MCT3/*SLC16A8*	0.302 ± 0.031^c^	0.269 ± 0.052^c^	0.615	0.893	Equal abundance
Monocarboxylate transporter 4	MCT4/*SLC16A3*	17 ± 3.9	17.2 ± 2.6	0.969	1.01	Equal abundance
Reduced folate transporter	RFC1/*SLC19A1*	1.02 ± 0.07	1.39 ± 0.17	0.0929	1.36	Equal abundance
Organic anion transporter 3	OAT3/*SLC22A8*	0.239 ± 0.027^c^	0.25 ± 0.063^c^	0.877	1.05	Equal abundance
Proton‐coupled folate transporter	PCFT/*SLC46A1*	1.89 ± 0.09	1.74 ± 0.27	0.631	0.924	Equal abundance
Multidrug and toxin extrusion protein 1	MATE1/*SLC47A1*	0.963 ± 0.082^c^	1.17 ± 0.05	0.0752	1.22	Equal abundance
Sodium‐potassium ATPase	Na^+^/K^+^ ATPase	20.1 ± 1.1	28.1 ± 3.8	0.0895	1.4	Equal abundance

^a^ Apical and basolateral membrane fractions, *n* = 3–4.

^b^ Evaluation based on apical/basolateral expression ratio: more than 1.5‐fold differences were considered as enrichment. Student's *t*‐test (two‐tailed) was used to generate the *p* values evaluating the difference in expression levels between apical and basolateral membranes were analyzed unpaired.

^c^ Some values that were located outside the standard curves were used for the calculation of the expression values.

### 3.3 Proteomics of drug‐metabolizing enzymes

The ocular tissues express a dramatically different profile of drug‐metabolizing enzymes as compared to the liver. For example, the main hepatic drug‐metabolizing cytochrome P450s (CYPs) including CYP isoforms 1A2, 2B6, 2C9, 2C9, 2D6, and 3A4 are expressed at the mRNA level very weakly if at all in human cornea, iris‐ciliary body and retina/choroid (Argikar et al., 2017; Nakano et al., 2014). In many cases, conflicting findings with regard to individual P450s and tissues have been reported (Dumouchel et al., 2018; Wolf et al., 2022). In contrast, other ocular CYPs such as CYP1B1, CYP4V2, and CYP4B1 seem to have a more specific role in pathology and physiology of ocular diseases (Nakano et al., 2014). In line with the above, global proteomics studies indicate the absence of main drug‐metabolizing CYPs but indicate the presence of, for example, CYP1B1, CYP20A1, CYP2A7, CYP4V3, CYP2F1, and CYP2J2 in cornea, iris‐ciliary body, vitreous, choroid, optic nerve, retina, and choroid/RPE (Ahmad et al., 2018). Similarly, the profile of other oxidative and conjugating enzymes in ocular tissues seems limited apart from glutathione S‐transferases important in elimination of reactive compounds (Dumouchel et al., 2018).

Ocular carboxylesterase (CES) isoforms have been investigated (Hammid et al., 2021, 2022) by measuring the enzyme activities and absolute protein quantities using mass spectrometry and targeted proteomics, respectively. For quantitative proteomics, an isotope‐labeled standard peptide for main CES isoforms was used for the first time to analyze preclinical (rabbits, pigs) and human post‐mortem ocular samples (Hammid et al., 2021, 2022).

Esterase activities towards various substrates have been found earlier in several ocular tissues in multiple species (Argikar et al., 2017; Dumouchel et al., 2018; Lee, 1983). Esterase activity has been utilized for the topical ophthalmic pro‐drug approach to improve corneal absorption of lipophilic pro‐drugs that are rapidly transformed into the active drug in the cornea (Ellis et al., 1972; Lee & Li, 1989; Mandell et al., 1978). Prodrugs with ester groups improve the lipophilicity and permeation of the compound to the cornea. Dipivefrin was the first ophthalmic prodrug that enhanced the ocular absorption of epinephrine by one order of magnitude (Mandell et al., 1978). Latanoprost, the drug of choice in glaucoma treatment, is an ester prodrug that is hydrolyzed to an active prostaglandin following topical ocular administration (Sjöquist & Stjernschantz, 2002). Moreover, an increase in the ocular absorption of many other ocular drugs has been observed with the ester prodrug approach (Ellis et al., 1972; Heikkinen et al., 2020; Macha et al., 2004; Volotinen et al., 2011).

Even though the presence of esterase proteins in ocular tissues has been detected by global proteomics (Ahmad et al., 2018), there is limited data available on the absolute expression levels of ocular esterases. Interestingly, the human CES1 levels among ocular tissues range around 10‐fold, with the highest contents in the retina and iris‐ciliary body (~2 pmol/mg protein), while the cornea had the lowest expression of ~0.2 pmol/mg (Hammid et al., 2022). CES2 is detectable at much lower levels (<0.1 pmol/mg) in human conjunctiva, cornea, retina, and RPE‐choroid (Hammid et al., 2022). Rabbits showed significant differentiated levels of CES1 and CES2 expression in ocular tissues: the highest concentration in the cornea and the lowest in the conjunctiva for CES1, while the opposite order was true for CES2. In pigs, CES1 was detected in most pig tissues while CES2 was not detected due to the absence of the CES2 gene in this species (Hammid et al., 2021).

Moreover, the esterase activity was investigated by following the pharmacokinetics of cefuroxime axetil prodrug, which is esterase substrate (for CES among others). The pharmacokinetic study was done after direct injection into the anterior chamber and vitreous cavity of the eye of rabbits and overall hydrolysis to cefuroxime was analyzed with LC/MS in ocular tissues (del Amo et al., 2022). These results suggest that a major part of cefuroxime axetil is eliminated via metabolism instead of other mechanisms. The highest cefuroxime levels were determined in the cornea, iris‐ciliary body, and aqueous humor indicating a high rate of hydrolysis in these tissues (del Amo et al., 2022). These in vivo findings follow a similar trend to the observed in vitro tissue activities using *p*‐nitrophenyl acetate (NPA), a generic esterase substrate (Hammid et al., 2021; Heikkinen et al., 2018), and with the CES1 content (Hammid et al., 2021). Other ocular esterases such as arylesterases, cholinesterases, and paraoxonases have been detected by global proteomics (Ahmad et al., 2018) but their expression levels have not been quantitated, and their role in prodrug activation is not known.

The studies indicate that there are remarkable differences in ocular carboxylesterase expression and activity among tissues and species. This should be taken into consideration in the design of ester prodrugs and drug conjugates, in the evaluation of ocular effects of systemic drugs, and translational and toxicity studies. Due to low levels of many other drug‐metabolizing enzymes in the eye, sophisticated techniques with sample enrichment and more sensitive detection methods are needed for a more comprehensive view of ocular drug metabolism. Overall, emerging data on ocular enzyme expression and (pro)drug metabolism, as well as species differences, facilitate translational development of ophthalmic drugs and prodrugs.

### 3.4 Future developments: Spatial proteomics in the ocular tissues

**Figure 4:**
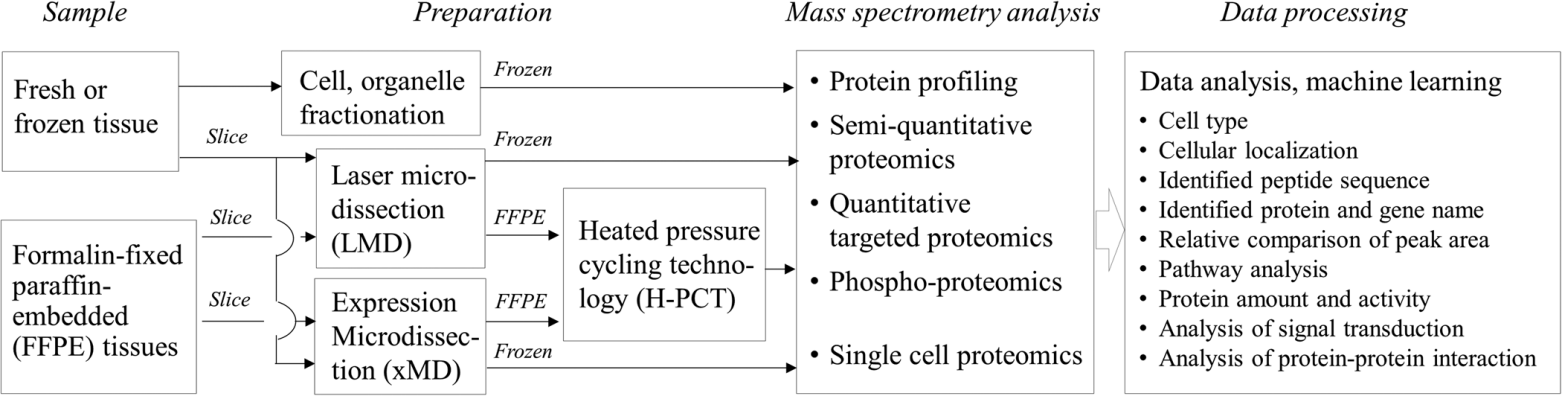
Scheme of the spatial proteomics in ocular tissues.

One of appealing future goals in ocular proteomics is to construct quantitative protein expression map in healthy and diseased human ocular tissues. It is also important to analyze the regulation mechanism of the transport function. Phospho‐proteomics will provide a useful technology for the analysis of signal transduction mechanism involved with barrier functions, for example, the oxidative stress‐mediated suppression of efflux transport function of MDR1 was revealed for the rapid internalization of MDR1 by phosphorylation of tyrosine‐14 in caveolin‐1 via Abl kinase and Src kinase pathway (Hoshi et al., 2020). Analysis of protein–protein interaction is one of the most challenging subjects in future ocular proteomics. Cross‐linking mass spectrometry is useful methodology for the discovery of receptor proteins to be interacted with ligand protein (Kuroda et al., 2019). The protein co‐fractionation coupled to mass spectrometry (CoFrac‐MS) technology will be the key technology for the analysis of protein–protein interaction (Salas et al., 2020). Such advance would have significant impact on understanding of physiology and pharmacology of ocular barriers. Furthermore, such data would provide rational insights to ocular drug discovery and development. As the ocular anatomy includes complex structures with different cell types, the development of quantitative protein map requires: (1) a reproducible method to pretreat formalin‐fixed paraffin‐embedded tissue and (2) a useful method to collect the cells of interest. The scheme in Figure 4 proposes a future workflow for spatial proteomics of ocular tissues.

*Heated pressure cycling technology (H‐PCT) for sample pretreatment of formalin‐fixed paraffin‐embedded (FFPE) tissues*. Availability of fresh or frozen human ocular tissues is extremely limited, whereas formalin‐fixed paraffin‐embedded (FFPE) tissue samples are widely available. The FFPE samples can be preserved at room temperature at least for several years. As formalin fixation induces crosslinking of proteins, efficient sample pretreatment is needed before FFPE samples can be used for the proteomics. The hydrostatic pressure cycling technology (PCT) has been established for a detergent‐free sample preparation technique for systems biology studies (Gross et al., 2008). In PCT, hydrostatic pressure forms an interdigitated state of lipid bilayer, while rapid release of the hydrostatic pressure destabilizes molecular interactions of lipids and membrane proteins, leading to efficient solubilization of the components. Zhu et al. reported that PCT‐assisted sample processing at 30°C followed by SWATH‐MS analysis of FFPE tissue samples gave protein expression levels that correlated with those of frozen tissue samples (Zhu et al., 2019). To facilitate de‐crosslinking and extraction of proteins from FFPE samples, the temperature of PCT was increased to 95°C. Previously used for FFPE sample solubilization would react with proteins at high temperatures. Therefore, urea was changed to phase‐transfer surfactant (PTS) buffer that is resistant at heated conditions and solubilizes proteins more effectively than urea. The heated PCT method proved efficient and reproducible in protein extraction from FFPE samples, enabling reliable quantitative proteomics for FFPE tissue sample with significantly improved accuracy (Uchida, Sasaki, et al., 2020).

*Laser‐capture microdissection and expression microdissection for the isolation of target cells*. Laser‐capture microdissection (LMD) is a well‐known method of tissue dissection that became popular in the analysis of heterogenous protein localization and proteomic expression studies (Tachikawa et al., 2018; Wang et al., 2008; Xiang et al., 2023). For example, heterogeneous drug metabolism in the liver is analyzed with this method (Katz, 1992; Takai & Tanaka, 2015). Cytochrome P450 enzymes are found in the pericentral (PC) vein hepatocytes, whereas the periportal (PP) vein hepatocytes have predominant role in the uptake and the biliary excretion of bile acids (Gumucio et al., 1978) and organic anions (Groothuis et al., 1982). After the PP and PC vein hepatocytes were dissected separately by LMD from mouse liver tissue slices, the proteins (CYP P450, transporters) were quantified by mass spectrometry (Tachikawa et al., 2018). The results demonstrated predominant expression of sinusoidal membrane transporters (e.g., organic anion transporting polypeptide 1a1 [Oatp1a1/*Slc21a1*], organic anion transporting polypeptide 1b2 [Oatp1b2/*Slc21a10*], organic cation transporter 1 [Oct1]) in the PC vein regions whereas the level of organic anion transporter 2 (Oat2) was greater in the PP vein regions. Interestingly, no significant zonation effect of ABC transporters (e.g., bile salt export pump [Bsep/*Abcb11*], Bcrp, multidrug resistance‐associated protein 2 [Mrp2/*Abcc2*]) were observed. Accordingly, LMD provides valuable spatial information on pharmacokinetically important proteins (transporters, enzymes), potentially facilitating understanding of heterogeneous and intra‐tissue distribution of drugs in healthy and diseased ocular tissues. LMD enables precise dissection of the target region from the tissue slice, but this method requires expertise, and it has only low throughput (Walsh & Halushka, 2023).

To overcome the limitations of LMD, the expression microdissection (xMD) method was developed by Tangrea et al. (2004). In this procedure, there are five steps: (1) the target cells are treated by an antibody that selectively binds to the cellular antigens in the tissue slice, (2) secondary peroxidase‐labeled antibody and chromogen substrate are added, (3) after incubation the tissue slice is covered with an polyethylene vinyl acetate (EVA) film, (4) the stained cells convert light energy to heat, melting the EVA film selectively and bind to the film, (5) the EVA bound cells are collected for the later analysis (Walsh & Halushka, 2023). The xMD is operator‐independent, high‐throughput, and low‐cost method. However, low availability of antibodies or other molecular tags for specific binding to the target cells limit the scope of this method in spatial ocular proteomics.

## 4 ANALYSIS OF DRUGS AND METABOLITES IN OCULAR SAMPLES

### 4.1 LC‐MS in ocular drug analysis

LC‐MS and SRM mode have been the method of choice in drug distribution studies since the late 1990s (Lee & Kerns, 1999). In addition to the typical pharmacokinetic studies with individual drugs, the LC‐MS methodology is well suited for cassette dosing methods in ocular pharmacokinetic studies, where an array of drugs is administered simultaneously (Fayyaz et al., 2020; Ramsay et al., 2017). The application of selected substrates and analysis of the metabolites can also be used to study the activity of metabolizing enzymes in ocular tissues (del Amo et al., 2022).

The most common instrument for drug quantitation is the triple‐stage quadrupole mass spectrometer due to its speed, ruggedness, sensitivity, wide linear range, and relatively low price. The separation methods are usually based on the use of reversed‐phase columns, formate or acetate buffers, and methanol or acetonitrile as the organic modifier. Table 5 lists a selection of articles, where LC‐MS has been used for drug analysis from solid and aqueous ocular tissues.

**Table 5:** Applications of liquid chromatography–mass spectrometry in ocular research.

Administration route for in vivo studies	Tissue/buffer	Analytes	Homogenization	Extraction	Chromatography	LC‐MS	Calibration range	Internal standard	Reference
Topical	Rabbit meibomian glands, conjunctival epithelium, palpebral conjunctiva, lid margin	Azitromycin	Bead mill homogenizer (Shake Master Auto) with water	PPT acetonitrile	XBridge Phenyl, 0.1% FA‐acetonitrile	QQQ Quantum Ultra	ND	Erythromycin	Asano et al. (2017)
Topical	Rabbit, monkey, aqueous humor, vitreous, retina, choroid	Brimonidine, dexamethasone	ND	LLE with ethyl acetate (dexamethasone rabbit aqueous humor); PPT 50% acetonitrile/50% methanol (both drugs monkey aqueous humor); 95% methanol/5% water (other tissues)	Atlantis T3 C18, 0.1% FA‐methanol	Qtrap Sciex 5500	0.1–1000 ng/mL for aqueous humor	Brimonidine‐d4 dexamethasone‐d4	Shen et al. (2014)
Topical	Rabbit cornea, retina‐choroid	Irbesartan, candesartan	Homogenization in diluted rabbit plasma	SPE, HLB cartridges	ACE Excel 5 SuperC18, 0.25% FA‐methanol	QQQ Agilent 6490	2–2000 ng/g	Candesartan‐d4 irbesartan‐d4	Tan et al. (2020)
Topical	Rabbit anterior, equatorial and posterior sclera, cornea, ciliary body, conjunctiva, iris, crystalline lens, retina	Atropine	ND	ND	SymmetryShield RP8, 0.02 M FA acetonitrile	ND, Single nominal res	ND	ND	Wang et al. (2019)
Topical, intracameral	Rabbit corneal epithelium, corneal stroma with endothelium, bulbar conjunctiva, anterior sclera, aqueous humor, iris‐ciliary body, lens, and vitreous humor	Betaxolol, timolol, atenolol	Corneal epithelium: lysis in 0.1 N NaOH. Other tissues: Bead mill homogenizer (Omni) in PBS	PPT acetonitrile, 1% FA	Poroshell 120 SB‐C18, 0.1% FA‐methanol	QQQ Agilent 6495	0.5–4000 nM	Atenolol‐d7 betaxolol‐d5 timolol‐d5	Fayyaz et al. (2021)
Topical micellar formulation	Rabbit aqueous humor, cornea, iris‐ciliary body, sclera, retina‐choroid	Dexamethasone, prednisolone	ND	PPT 20% (v/v) perchloric acid followe by LLE with TBME	Phenomenex C18, isocratic, 0.1% FA acetonitrile	QTRAP Sciex 2000	2.70–617 ng/mL for dexamethasone	Hydrocortisone	Earla et al. (2010)
Topical nanomicellar formulation	Rabbit cornea, iris ciliary body, lens, sclera, retina‐choroid, vitreous, aqueous humor	Rapamycin	Tissue homogenizer (Tissue Tearor), phosphate buffer (pH 7.4)	50% triethyl amine in methanol, followed by methanol	C 8 Xterra, Isocratic, 0.05% FA acetonitrile	QTRAP Sciex 3200	2.3–1000 ng/mL	Erythromycin	Earla et al. (2012)
Topical ophthalmic gel	Rabbit cornea, conjunctiva, tear fluid, aqueous humor	Lidocaine	High‐speed homogenizer (ND), 0.9% NaCl	PPT acetonitrile	Hedara ODS‐2, isocratic0.1% FA. methanol with 20 mM ammonium acetate	QQQ Agilent 6410B	0.05–60 ng/mL for plasma, 2.5–2,500 ng/mL for cornea	Letrozole	Liu et al. (2015)
Topical suspension with and w/o cyclodextrin	Rabbit vitreous, aqueous humor, retina	Indomethacin	Retinal samples homogenized in ammonium acetate buffer (20 mM, pH 7.2) (homogenizer ND)	LLE with ethyl acetate	Phenomenex Gemini‐NX, eluent ND	QQQ Agilent 6410	1–200 ng/g for retina	Diclofenac	Bucolo et al. (2011)
Topical suspension	Rabbit tears, aqueous humor, conjunctiva, cornea	Besifloxacin	ND	PPT methanol	Sepax GP‐Phenyl, Isocratic, methanol–acetonitrile‐5 mM ammonium formate–formic acid	QQQ Quantum Ultra	2.06–2060 ng/mL	Nateglinide	Gu et al. (2016)
Topical sustained release gel	Rabbit cornea, aqueous humor, vitreous	Cysteamine	Cornea cryopulverized in liquid nitrogen with a stainless steel pulverizer, followed by bead beating homogenizer (FastPrep 24)	PPT 95% acetonitrile/1% formic acid	X‐Bridge amide, isocratic 80/20 acetonitrile/water, with 0.1% formic acid and 2 mM ammonium formate	QQQ Sciex 4500	15 nM−4000 nM	Cysteamine‐d4	Jimenez et al. (2022)
Intracameral, intravitreal	Rabbit cornea, iris‐ciliary body, lens, vitreous humor, neural retina	Acetaminophen, sunitinib, brimonidine, cefuroxime, acetaminophen‐sulfate, N‐desethyl sunitinib, cefuroxime, and brimonidine‐2,3‐dione	Bead‐beating homogenizer (Precellys Evolution)	PPT ethanol:water mixture. Cornea: TMBE extraction	Luna Omega C18, 0.1% FA‐acetonitrile	QQQ Sciex 5500+	0.05–10,000 nM	Brimonidine‐ d4, cefuroxime‐d3 sunitinib‐d10, acetaminophen‐d4	del Amo et al. (2022)
Intracameral	Rabbit aqueous humor	Betaxolol, timolol, atenolol		PPT acetonitrile, 1% FA	Poroshell 120 SB‐C18, 0.1% FA‐methanol	QQQ Agilent 6495	2.5−2000 nM for atenolol and timolol 10−2000 nM for betaxolol	Atenolol‐d7 betaxolol‐d5 timolol‐d5	Fayyaz et al. (2020)
Intravitreal implant	Rabbit aqueous humor, vitreous, retina, choroid‐RPE	Fluocinolone acetonide	ND	chlorobutane and chloroform (90:10)	YMC ODS‐A C18, isocratic acetonitrile, 10 mN ammonium acetate, methanol	QQQ Sciex 3000	200–51,200 pg/mL	Triamcinolone acetonide‐d7	Kane and Green (2015)
Intravitreal and subtenon	Rabbit cornea, iris, lens, vitreous, retina, choroid	Prinomastat	ND	PPT acetonitrile/methanol	Zorbax XDB C18, eluent ND	QQQ Micromass Quattro II	ND	AG3340–d8	Cheng et al. (2001)
Sub‐tenon drug delivery system, subconjunctival and intravenous injections	Rabbit cornea, iris, lens, retina‐choroid, aqueous humor, vitreous, sclera	Dexamethasone	Homogenized in 0.9% NaCl (homogenizer ND)	n‐hexane and acetate ester (1:1, v/v)	Hypersil‐Hypurity C18, Isocratic, 5 mM ammonium formate, methanol, acetonitrile	Single Q Shimadzu LC‐MS 2010	ND	Triamcinolone	Huang et al. (2017)
Subconjunctival implant	Rabbit sclera, conjunctiva, cornea, aqueous humor, vitreous	Ilomastat	Lyophilization, followed by Incubation with Proteinase K	PPT methanol, diethyl ether was added to precipitate fatty acids. Ethyl acetate extraction.	Zorbax Eclipse Plus C18, 0.1% FA‐acetonitrile	Ion trap LCQ	62.5–1000 pg/20 mg of cornea	Marimastat	Mohamed‐Ahmed et al. (2018)
Oral	Rat cornea, iris‐ciliary body, lens, neural retina, RPE‐choroid, sclera	Dexamethasone, Levofloxacin, BI1026706, BI113823	Bead‐beating homogenizer (Precellys Evolution)	PPT 100 mM NH4OH in Ethanol:water (4:1) and Ethanol:water (4:1)	Acquity HSS T3 (BI compounds), Luna Omega Polar C18 (dexamethasone, levofloxacin), eluent ND	QQQ Sciex 6500	0.05‐500 nM	[13C2D2]BI113823, [13CD215N]BI1026706	Rimpelä et al. (2020)
Ophthalmic artery catheterization	Rabbit vitreous, retina	Melphalan	ND	PPT acetonitrile	ND	QQQ Quantum Ultra	ND	Carbamazepine	Daniels et al. (2018)

In vitro studies	Tissue/buffer	Analytes	Homogenization	Extraction	Chromatography	LC‐MS	Calibration range	Internal standard	Reference
Conjunctival permeability (Using/diffusion chamber)	Porcine conjunctiva/BSS Plus buffer	Cassette mix containing 34 drugs		Filtration	Poroshell 120 SB‐C18, 0.1% FA‐methanol	QQQ Agilent 6495	0.1 −500 ng/mL	Atenolol‐d7, atropine‐d5, cephalexin‐d5 dexamethasone‐d5, fluconazole‐d4, ganciclovir‐d5, indomethacin‐d4, lincomycin‐d3, lornoxicam‐d4 methotrexate‐d3	Ramsay et al. (2017)
Partition into lens (incubation)	Porcine lens	Cassette mix containing 32 drugs	Plastic homogenization pestles and ULTRA‐TURRAX in HBSS–HEPES buffer	PPT acetonitrile	Poroshell 120 SB‐C18, 0.1% FA‐methanol	QQQ Agilent 6495	ND	Atenolol‐d7 atropine‐d5 fluconazole‐d4 lincomycin‐d3	Heikkinen et al. (2019)
Analytical method development (spiked tissue)	Rabbit cornea, aqueous humor	Cassette mix containing 27 drugs	Glass‐glass homogenizer (cornea in PBS), freeze‐thaw lysis	PPT acetonitrile/methanol	kinetex C18, 0.1% FA‐acetonitrile	QTrap Sciex 4500	40–4800 fmols/10 µL injection	Dexamethasone‐d4, fupirtine‐d4, timolol‐d5	Matter et al. (2022)

Abbreviations: ESI, electrospray; FA, formic acid; LLE, liquid‐liquid extraction; ND, not defined; PPT, protein precipitation; RPE, retinal pigment epithelium; TBME, tert‐butylmethylether.

Before the LC‐MS analysis, the eye must be dissected to distinct tissue samples, representing cornea, conjunctiva, sclera, aqueous humor, iris, lens, retina, and vitreous. Samples from aqueous humor and vitreous can be directly prepared using methods commonly used for serum or plasma samples. The other tissues must be homogenized with specific methods depending on their structure and consistency. During the dissection, extra care must be taken to avoid cross‐contamination from the tissues with high drug concentrations, especially as parts of the sample are very fluid and brittle (Fayyaz et al., 2021; Mori et al., 2019). The homogenization can be done by a bead‐beating homogenizer or manually in liquid nitrogen, in some cases lysis with proteinase, sodium hydroxide or repeated freeze‐thaw cycles have been used to break the cells (Table 5). The more fibrous tissues, like the cornea and sclera, must be cut with a scalpel before the use of a bead homogenizer. The workflow of ocular sample preparation with special emphasis on melanin binding has been well described by Rimpelä et al. (2020).

Even though LC‐MS is a very selective and sensitive method, it is significantly affected by signal suppression that is caused by the co‐eluting matrix molecules (“the matrix effect”) (Van Eeckhaut et al., 2009). The limit of quantitation depends on the analyte and the ocular tissue type and may vary by more than two orders of magnitude (del Amo et al., 2022). This effect is caused mainly by phospholipids and salts, and as it is dependent on the tissue type, the suitability of the extraction method must be evaluated separately for each sample matrix. The most common extraction methods used for ocular sample preparation for LC‐MS are protein precipitation (PPT), liquid–liquid extraction (LLE), and solid phase extraction (SPE). The PPT with acetonitrile is the most straightforward extraction method, but it does not effectively remove the matrix effect that is due to the lipids in the sample. Sample preparation by LLE needs more manual work and time, but with careful selection of the extraction solvent and conditions rather clean extracts can be obtained. The use of SPE also results in very clean samples, but the recovery for different analytes varies. This may cause problems especially if the analytes in a cassette mix have a wide range of physico‐chemical properties. More selective SPE and LLE methods provide a possibility to concentrate the samples by evaporation. To compensate for variations in extraction yield and LC‐MS signal, the use of isotopically labeled internal standards is a widely accepted preferred practice in drug analysis (Adaway & Keevil, 2012; Henion et al., 1998; Rappold, 2022; Van Eeckhaut et al., 2009).

Administration of a cassette mix of several drugs in the same rabbit experiment enables pharmacokinetic experiments with fewer animals. For example, a comparative rabbit study of five drugs with six‐time points with data from five eyes at each sampling time requires 150 rabbits if one eye of each rabbit is dosed, and 75 rabbits if both eyes are used. Simultaneous administration of a cassette mix of five compounds can be performed with 15 rabbits if the drug application is done to both eyes of the rabbits. This approach relies on LC/MS analytics and is in accordance with the 3R principle in animal testing (reduce, refine, replace). Furthermore, this approach reduces inter‐occasion on interindividual variability in the experimental data, and this way reveals more reliably the pharmacokinetic differences between the test compounds.

### 4.2 Mass spectrometry imaging in ocular research

Because drug penetration and distribution to the eye are hindered by physical and biological barriers and drug effects are mediated by specific cell populations within the tissues, it is important to study the drug distribution at a fine structural level. As described in the previous chapter, it is possible to dissect ocular tissues and analyze the drug contents by LC‐MS. However, this process is cumbersome, dissection of the tissues at fine levels is difficult, and the number of samples increases dramatically if detailed information on compound localization is needed. Thus, it is a very tempting idea to use molecular imaging to collect data from thousands of data points within an ocular cross‐section at detailed intra‐tissue level. This approach also enables analyzes from human tissue sections and rodent eyes that are particularly challenging for dissection. Another advantage is the co‐localization of drug with cell‐specific marker compounds, drug target molecule, or some other compound of interest.

There are several imaging technologies suitable for pharmacokinetic studies, such as optical imaging including fluorescence and infrared analysis, autoradiography, radionuclide imaging, and mass spectrometry (Ban et al., 2022). The most important noninvasive methods for ocular imaging are optical coherence tomography (OCT) and MRI. These methods can be used to a certain extent also for the measurement of the spatial distribution of drugs and formulation excipients in the eye (Kopp et al., 2017). However, they do not inform us about the exact chemical structure of the detected material and their sensitivity and linearity are far from optimal.

Mass spectrometric imaging (MSI) provides an opportunity to determine tissue localization of the analytes without the use of radio‐ or fluorescent‐labeled compounds. The common MSI techniques for drug analysis as well as the limitations and advantages of these methods have been extensively reviewed (Bodzon‐Kulakowska & Suder, 2016; Castellino et al., 2011; Chen, Xie, et al., 2022; Davoli et al., 2021; Nilsson et al., 2015). The review by Bowrey et al. (2016) gives a good overview of approaches applying MSI technology to ocular pathology, and the predicted future developments concerning resolution, coverage, and bioinformatics that are currently coming of age. The main advantage of MSI compared to other imaging methods is the possibility for simultaneous spatial analysis of several drugs, their metabolites, and endogenous compounds in biological samples. In addition to spatial analysis of drugs and their metabolites, it is possible to follow pharmacodynamics and toxicological effects by simultaneous analysis of endogenous biomarkers, which are most often lipids. In most cases, instrument with a high‐mass resolution instrument has been used for MSI (Table 6), because quantitative information on exogenous compounds is hard to obtain with unit‐resolution MSI (Yamada et al., 2018). In addition to high resolution, methods like ion mobility analysis (Spraggins et al., 2019) and on‐tissue MS/MS can be used to increase the specificity of detection (Boughton et al., 2020; Pereiro et al., 2020; Wang et al., 2019; Yamada et al., 2018). However, especially the use of MS/MS may decrease the speed of acquisition, because of the longer data collection times.

**Table 6:** Applications of mass spectrometry imaging in ocular research.

Tissue	MSI technology	Analytes	Administration	Spatial resolution	Quantitation	Normalization	Combination with other techniques	References
Human eye	MALDI LTQ MS/MS	Benzalconium chloride, lipids	Topical	120 µm		TIC		Garrett et al. (2011)
Human eye	MALDI Orbitrap	Phospholipids	Endogenic lipids	100 µm		TIC		Seng et al. (2018)
Human eye	MALDI tof or LA‐ICP‐MS	Lipids, Co, Zn, Fe	Endogenic, uveal melanoma	50 µm (MALDI) 150 µm (LA)		carbon‐13 (LA)	Microscopy	Cole et al. (2021)
Human and bovine lens	MALDI tof, MALDI tof/tof	Crystallin proteins	Endogenic proteins	300 µm			LC‐MS, proteomics	Anderson, Nye‐Wood, et al. (2020)
Human and bovine lens, rabbit retina	MALDI tof	Aquaporin, opsin	Endogenic	200 µm		TIC		Grey et al. (2009)
Human lens	MALDI Q‐tof, MS/MS	Sphingolipids	Endogenic	50 µm			LC‐MS	Deeley et al. (2010)
Human lens	MALDI tof	Crystallin fragments	Endogenic	60 µm		RMS	LC‐MS	Schey et al. (2022)
Human lens	MALDI tof, MALDI FT ICR	Aquaporin forms	Endogenic			TIC	LC‐MS	Wenke et al. (2015)
Human lens	MALDI FT ICR, MS/MS	Tryptophan metabolites	Endogenic	150 µm		RMS	LC‐MS	Demarais et al. (2019)
Human lens	DESI LTQ, LTQ‐Orbitrap	Lipids	Endogenic	200 µm	Relative		FIA, DESI‐MS/MS	Ellis et al. (2010)
Human optic nerve	MALDI tof	Lipids, proteins	Endogenic	20 µm		TIC	LC‐MS, proteomics	Anderson, Spraggins, et al. (2015)
Human retina	MALDI tof	A2E and metabolites	Endogenic	300 µm		Maximal A2E signal	Fluorescence microscopy	Ablonczy et al. (2013)
Human retina	MALDI FT ICR	Lipids	Endogenic lipids	10 µm		RMS	Fluorescence microscopy, LC‐MS	Anderson, Nye‐Wood, et al. (2020)
Human retina, frozen and PFA fixed	MALDI FT ICR	Lipids	Endogenic lipids	15 µm			LC‐MS	Kotnala et al. (2021)
Monkey eye	MALDI LTQ MS/MS	Lipids	Endogenic	80 µm		TIC	Ophthalmoscopy	Garrett and Dawson (2009)
Bovine lens	MALDI FT ICR	Glutathione conjugates	Endogenic	150 µm		RMS	LC‐MS	Nye‐Wood et al. (2017)
Bovine lens	MALDI FT ICR	Carbon‐13 glucose	Incubation	30 µm	Relative	Maximal signal	IHC, SDS‐PAGE, GC‐MS	Zahraei et al. (2022)
Sheep eye	SIMS‐tof	Dexamethasone	In vitro perfusion					Mains et al. (2011)
Sheep lens	nanoDESI Orbitrap	Aquaporin tetramer	Endogenic	200 µm		TIC	DESI‐MS/MS	Hale and Cooper (2022)
Porcine eye	MALDI FT ICR	Beta‐blocking drugs	Topical	50 µm	Relative	RMS	LC‐MS	Balla et al. (2021)
Porcine lens	MALDI FT ICR	Sphingolipids	Endogenic	150 µm	Relative	TIC		Vidová et al. (2010)
Porcine lens	MALDI FT ICR	Mix of 16 drugs	Incubation	150 µm	With isotope labeled IS	d5‐atropine	LC‐MS	Heikkinen et al. (2019)
Porcine lens	MALDI FT ICR	Phospholipids	Endogenic lipids	150 µm		TIC	LC‐MS	Pol et al. (2011)
Pig retinal sections, cells	MALDI Orbitrap	Lipids	Endogenic lipids	15 µm			IHC	Pereiro et al. (2020)
Rabbit eye	DESI LTQ	Azithromycin	Topical	150 µm	Standards pipetted on tissue for estimation		LC‐MS	Asano et al. (2017)
Rabbit eye	MALDI tof or MALDI tof/tof	Benzalconium chloride	Topical, long term	80 µm			IHC	Brignole‐Baudouin et al. (2012)
Rabbit eye	MALDI LTQ MS/MS	Melphalan	Intra arterial	150 µm	Standards pipetted on tissue for estimation		LC‐MS, angiography, microscopy	Daniels et al. (2018)
Rabbit eye	MALDI tof/tof	Brimonidine	Topical	80 µm	relative		MALDI MS/MS	Grove et al. (2021)
Rabbit eye	MALDI FT ICR	Atropine	Topical	10 µm		TIC		Mori et al. (2019)
Rabbit eye	MALDI FT ICR	23‐aa peptide Antivascularization drug	Topical	45 µm	Relative	RMS	OCT, microscopy, IHC	Obasanmi et al. (2022)
Rabbit, mouse and black bream eyes	MALDI FT ICR, MALDI tims‐tof	Small metabolites and lipids	Endogenic	30 µm FT ICR 10 µm tims‐tof		RMS		Boughton et al. (2020)
Rat eye	MALDI tof or MALDI tof/tof	Small metabolites and lipiids	Endogenic, dry eye disease	50 µm		TIC	Fluorescence microscopy LC‐MS	Chen, Zhang, et al. (2022)
Rat eye	MALDI LTQ MS/MS	BMS drug and metabolites	Oral	250 µm			Autoradiography	Drexler et al. (2011)
Rat eye	MALDI FT ICR	Retigabine and metabolites	Oral supplement	5 µm			Microscopy	Groseclose and Castellino (2019)
Rat eye	MALDI tof	Kinase inhibitor drug	IV infusion	10 µm	Calibration curve pipetted on tissue	TIC	Microscopy, LC‐MS	Hamm et al. (2022)
Rat eye	MALDI Q tof	Choloroquine	Oral	150 µm			MALDI MS/MS, microscopy	Yamada et al. (2011)
Rat and zebrafish lens	MALDI tof	Crystallin fragments	Endogenic	80 µm		TIC	Ophthalmoscopy	Anderson, Floyd, et al. (2015)
Rat retina, optic nerve	MALDI FT ICR	Sphingolipids	Endogenic lipids	25 µm			IHC	Fan et al. (2022)
Rat optic nerve	MALDI FT ICR	Lipid sulfatides	Endogenic	15 µm	Relative		microscopy, IHC	Stark et al. (2018)
Mouse eye	MALDI tof or LA‐ICP‐MS	Tryptic peptides from crystallin, histone proteins, leptin, Cu, Zn	Endogenic	25 µm MALDI	Calibration curve pipetted on gelatin for metals	TIC		Millar et al. (2022)
Mouse eye	DESI LTQ	Phospholipids	Endogenic after aptamer treatment	250 µm			Microscopy, IHC	Subramanian et al. (2016)
Mouse eye	MALDI tof/tof	Atropine	Topical	100 µm			LC‐MS	Wang et al. (2019)
Mouse eye	MALDI tof or MALDI Orbitrap	Anthocyanin glycosides	Intraperitoneal				MALDI MS/MS	Yamada et al. (2018)
Mouse retina, optic nerve	MALDI tof	Lipids, A2E	Endogenic	10 µm				Anderson et al. (2017)
Murine retina	DESI Orbitrap	Endogenous metabolites	Oral supplement	200 µm	Nontargeted metabolomics	TIC	LC‐MS, proteomics	Wert et al. (2020)
Mouse retina	MALDI tof	A2E and metabolites	Endogenic	150 µm		TIC	Fluorescence microscopy, MALDI LTQ MS/MS	Grey et al. (2011)
Mouse retina	MALDI tof	A2E and metabolites	Endogenic	150 µm		TIC	MALDI FT ICR MS/MS	Ablonczy et al. (2014)
Mouse retina	NanoSIMS	15 N/14 N ratio	15 N labeled diet	75 nm			Microscopy	Bonnin et al. (2021)
Zebrafish eye	MALDI iMSScope	Phospholipids	Fibronil in water	100 µm			Microscopy	Wenjie et al. (2020)

*Note*: When different resolutions were used, the lowest value is given.

Abbreviations: IHC, immunohistochemistry; LA‐ICP, laser ablation inductively coupled plasma; LC‐MS, liquid chromatography–mass spectrometry; MALDI, matrix‐assisted laser desorption/ionization; RMS, root mean square; TIC, total ion current.

MSI generates two‐dimensional maps of analyte intensities across the surface of a tissue. The term spatial resolution, or pixel resolution, generally refers to the center‐to‐center distance between two adjacent areas of acquisition (pixels).

Spatial resolution, which depends on step size, laser spot size, and stage stability, is one of the most important parameters that define the utility and scope of MSI as applied to tissue distribution studies (Castellino et al., 2011; Garrett & Dawson, 2009). Compromises between speed, resolution, and sensitivity are always necessary with MSI. Before each experiment, it is important to plan what resolution level is required for spatial separation of the structures of interest. Imaging with high resolution will lead to long acquisition times and loss of sensitivity as smaller amounts of material are analyzed: in a pixel of 100 µm × 100 µm the amount of analyte is four orders of magnitude higher than in a pixel of 1 µm × 1 µm. Vice versa, it would take 10,000 times longer time and more data storage to collect the data using the smaller resolution. The current performance of MSI with spatial resolution below 30 µm enables drug distribution analyzes at the cellular level. Boughton et al. (2020) have demonstrated that with 10 μm spatial data, it is possible to describe all major ocular tissue types in mice, including cornea (epithelium and stroma), iris, retinal layers, optic nerve bundle, and choroid/sclera. In this study, several typical marker compounds, mainly lipids, were found for each structure. An even more detailed study on the identification of lipids defining the retinal layers has been described by Anderson, Messinger, et al. (2020). Resolution of 5−10 µm allows to map drug distribution in the corneal (Mori et al., 2019) and retinal layers (Groseclose & Castellino, 2019). In the latter study lateral resolution was high enough to prove that the access of drugs to the eye was restricted by the blood‐retinal barrier.

There are several ion sources available for MSI imaging. Matrix‐assisted laser desorption ionization (MALDI) is the most often used technology due to the good spatial resolution needed for eye imaging, but also other methods such as desorption electrospray ionization (DESI) MSI has been used to study the distribution of dietary supplements and drugs in the eye at a spatial resolution of 150−200 µm (Asano et al., 2017; Wert et al., 2020). DESI has also been used to monitor therapeutic and toxic responses in ocular lipids at a resolution of 250 µm (Subramanian et al., 2016). Even though better spatial resolution can be achieved by MALDI, lower limit of drug detection can often be achieved with DESI imaging. The ion intensity, and the lower limit of drug detection, are dependent on the molecule‐specific ionization efficiency and the ionization method. DESI and MALDI are complimentary methods and one may be more useful for a certain classes of drugs than the other (Dannhorn et al., 2022). Very good spatial resolution, well below 1 µm, has been obtained using secondary ion mass spectrometry (SIMS) for retinal cell analysis. SIMS results in the heavy fragmentation of organic molecules and it is best suited for isotope analysis (Bonnin et al., 2021). However, the detection of drug molecules by SIMS is also possible if a compound‐specific fragment ion, such as fluoride anion (e.g., dexamethasone), can be detected (Mains et al., 2011). Another method suited mainly for the analysis of metals is laser ablation inductively coupled plasma mass spectrometry (LA‐ICP‐MS), used to detect copper, zinc, and iron linked to uveal melanoma (Cole et al., 2021), as well as copper and zinc in age‐related macular degeneration (Millar et al., 2022). However, this method has limited applicability in drug research.

In MALDI imaging sample preparation, typically fresh‐frozen tissues, are sectioned at 10–30 µm thickness. Step by step description of the procedure has been published by Anderson et al. (2017). For the cryo‐sectioning of small or sensitive samples, the selection of embedding material is crucial. The selected polymer for embedding should support the fragile structures, such as retinal layers, during sectioning at subfreezing temperatures. Also, the embedding process should not cause delocalization of analytes within the tissue, and it should not leak polymers, which alter the mass spectrometric signal or contaminate the instrument. For example, the most common, and otherwise excellent embedding material, the “optimal cutting temperature material”, tends to cause massive series of polyethylene glycol‐related ions, which disturb the signal, especially in full scan analysis mode. Alternative solutions include cutting the samples without any embedding material (Zahraei et al., 2022) or using less contaminating polymers such as gelatin (Wenjie et al., 2020), methylcellulose (Hamm et al., 2022) or poly,N‐(hydroxypropyl)‐methacrylamide (Dannhorn et al., 2020). Ocular samples can also be imaged by preparing a flat‐mount where the pigment epithelium is removed from the vitreous and flattened to the surface for analysis. By preparing the tissue in this manner, the migration of chemicals from anterior to posterior parts can be assessed for example after delivery of eye drops (Garrett & Dawson, 2009, Garrett et al., 2011).

The frozen sections are then mounted onto MALDI target plate, which are often conductive glass slides, before desiccation and application of the UV‐absorbing matrix. Due to high water content and brittleness, ocular tissues are easily damaged during cryo‐sectioning. Another typical feature in ocular drug analysis is the very wide concentration gradient across the sample. After topical ophthalmic administration, on the corneal surface, the typical drug concentration of the drop is >10 µg/mg, while the concentration in the retina may be a few ng/mg of tissue (Wang et al., 2019). To keep the structure intact and avoid cross‐contamination, a tape transfer method (Kawamoto, 2003) is often used to support the section during the process. Another critical step to decrease the delocalization, especially for highly water‐soluble analytes in the ocular sample preparation, is whether the sample is dried frozen, or at room temperature before matrix addition (Boughton et al., 2020).

One of the main issues of MALDI imaging is the very low MS signal for various analytes. As expected, neutral compounds, such as steroids, do not give a good signal under MALDI, but also analytes with readily ionizable groups may show rather poor sensitivity. In a study dealing with drug partitioning into the ex vivo porcine lens tissue (Heikkinen et al., 2019), only 11 out of 16 drugs gave acceptable signals when the standard solution was loaded on isolated lens section, and only five drugs (atropine, tizanidine, propranolol, pindolol, and pilocarpine) were successfully resolved from the background. The signal may be enhanced to some extent by washing the sample plate with chloroform to remove suppressive lipids, or with ammonium formate to remove salt adducts (Enthaler et al., 2013). However, in some cases, the formation of adduct ions can even be beneficial, for example, when neutral sugars are analyzed as chloride adducts (Boughton et al., 2020; Zahraei et al., 2022). Another strategy to improve the detection of poorly ionizable compounds is the chemical derivation, for example, tagging the molecules with easily ionizable groups (Harkin et al., 2022; Källback et al., 2020). However, both methods, washing, and derivatization, may lead to the delocalization of the analytes. This is problematic, especially in ocular drug imaging, where steep drug concentration gradients are typical, and major parts of the tissue are highly aqueous with low drug binding capacity.

With MSI, the relative and absolute quantitation suffers markedly from ion competition and suppression caused by endogenous compounds, mainly lipids, and salts. In addition to low ionization yield of drugs, the main problem is the uneven signal that is caused by variable ion suppression in non‐homogenous tissues. However, quantitative results obtained with MSI may correlate well with data measured by conventional LC‐MS methods (Deeley et al., 2010; Källback et al., 2020; Reyzer et al., 2003), Dannhorn et al., 2022) and by autoradiography (Khatib‐Shahidi et al., 2006). Signal intensity normalization and the use of isotope‐labeled standards can enhance the quantitation (Grey et al., 2021; Källback et al., 2020), and at the very least, MSI can generate qualitative data on drug distribution within the tissue (Asano et al., 2017; Daniels et al., 2018; Wang et al., 2019). The calibration curve is often generated by spotting a range of standards on blank tissue. The resulting concentration in the tissue can be calculated by accounting the droplet area and tissue thickness. A more sophisticated method uses a mimetic model, where the analyte standards are mixed with a tissue homogenate, frozen, cut, and mounted on the target plate (Groseclose & Castellino, 2013). Both these sample preparation methods have been shown to give reproducible results in a multicenter drug quantitation study (Barry et al., 2019). In addition, to their use of internal standards for absolute quantitation, stable isotopes can also be utilized for the analysis of metabolic flux and signal normalization (Grey et al., 2021). In the study of Zahraei et al. (2022), this kind of MSI fluxomics was applied to study glucose uptake and metabolism in the bovine lens.

The exact localization of the drug distribution is possible by co‐localization with cellular lipid biomarkers (Balla et al., 2021) or other tissue‐specific compounds such as *N*‐retinyl‐*N*‐retinylidene ethanolamine (A2E) found in the RPE (Grey et al., 2011; Groseclose & Castellino, 2019). Because MSI or even MS/MS data is not always selective enough for identification, microdissection combined with LC‐MS/MS is often used for verification of the compound identity (Chen, Zhang, et al., 2022). Analysis of the samples with microdissection and LC‐MS can be used to support MSI results or used as the sole method for quantitation (Daniels et al., 2018; Dilillo et al., 2017). Despite some issues in quantitation, MSI methods can provide unique information on e.g. uneven distribution of drugs in the cornea after topical ophthalmic administration, which is very tedious and cumbersome to detect with dissection methods (Balla et al., 2021).

The drug distribution data can be combined with biomarker data obtained by MSI or other imaging methods. When spatial effects of drug treatment on proteins have been studied, MSI images have mostly been combined with microscopy, immunohistochemistry (IHC), or microdissection data. An ideal way to combine molecular images with anatomical data is co‐registration with widely used hematoxylin‐eosin‐stained images to see the effects of the treatment (Castellino et al., 2021; Cole et al., 2021; Yamada et al., 2011). Another good example of the integration of different imaging methods is the study by Hamm et al. (2022), where using MSI and IHC images for successive tissue sections revealed a correlation between inflammatory areas with benzalkonium chloride localization (Brignole‐Baudouin et al., 2012).

Most MSI applications in ocular research deal with lipid analysis (Table 6), due to their high concentration and good ionization properties. It is expected that the number of pharmacokinetic studies using MSI will rapidly rise with technical developments. The addition of ion mobility separation device between the ion source and mass analyzer will enable simultaneous analyzes of isomeric compounds. Another technical improvement to increase the sensitivity of MALDI for several drugs is the use of a second laser for post‐ionization of the sample plume generated with the first laser, the so‐called MALDI‐2 (Dreisewerd et al., 2022). So far, the specificity of MSI is rather limited for spatial proteomics, but the technology has been successfully used for analysis of crystallin fragments, aquaporin forms, and other abundantly expressed proteins (Anderson, Floyd, et al., 2015; Anderson, Nye‐Wood et al., 2020, Millar et al., 2022; Schey et al., 2022; Wenke et al., 2015, Table 6). The spatial analysis of drug transporter proteins by MSI has not been possible, but more abundant membrane proteins, such as aquaporins have been successfully imaged by MALDI from lens tissue after denaturation (Grey et al., 2009), or directly as tetramers using nano‐DESI (Hale & Cooper, 2022).

## 5 SUMMARY

Mass spectrometry has been proven an excellent tool in ocular drug research, where small sample sizes and low concentrations are typical. Global and quantitative target proteomics give rational insights into pharmacokinetics, pharmacodynamics, and toxicokinetics by analyzing protein level, and posttranslational modification in the target region. The sensitivity and speed of LC‐MS make simultaneous quantitation of various drugs possible in minute tissue samples, even though ocular sample preparation needs extra care due to tissue properties and highly variable concentration gradients in the sample. The MSI methodology is on the verge of becoming as important as LC‐MS in ocular pharmacokinetic studies, since the spatial resolution has reached a level, where cell layers can be separated, and quantitation with isotope‐labeled standards has come more reliable. Mass spectrometry will remain in the foreseeable future as the main analytical method that will progress our understanding of ocular pharmacokinetics and, in selected cases, also pharmacodynamics.

## AUTHOR CONTRIBUTIONS

**Eva M. del Amo**: Visualization; writing—original draft; writing—review and editing. **Kati‐Sisko Vellonen**: Writing—original draft; writing—review and editing. **Arto Urtti**: Writing—original draft; writing—review and editing. **Tetsuya Terasaki**: Visualization; writing—original draft; writing—review and editing. **Anam Hammid**: Writing—original draft; writing—review and editing. **Paavo Honkakoski**: Writing—original draft; writing—review and editing. **Seppo Auriola**: Writing—original draft; writing—review and editing.

## CONFLICT OF INTEREST STATEMENT

The authors declare no conflict of interest.
